# Surgery

**Published:** 1893-10

**Authors:** 


### SURGERY.

EDITED BY DR. F. W. MCRAE.

### DIAGNOSIS OF HEREDITARY SYPHILIS.

Fournier insists that the diagnosis of hereditary syphilis must be based almost exclusively upon ocular evidence. Beyond a family history of infantile polymortality, verbal information has but little significance. To Hutchinson's triad of symptoms—notched teeth, interstitial keratitis, and inflammation of the middle ear—must be added three other important evidences of specific trouble that do not always receive sufficient emphasis. First, the general appearance of the patient; next, the cicatrices upon the skin and mucous membranes; and third, the deformities of the bones. Persistent childhood best describes the bodily condition, owing to the low stature and slight frame. The scars upon the skin and mucous membranes present four definite characteristics: their size, configuration, area and locality. They are very small, almost minute; in form like a rounded bow, an arcade, or a serpentine curve. They are arranged in groups joined at the side, and are found at the commissure of the lips, about the circumference of the nose, in the throat, and particularly upon the buttocks and in the lumbar region. In the bony skeleton are the most strongly marked, the most profound and persistent lesions, easily discovered in the skull, in the face, in the bones of the extremities, most notably in the tibia. The forehead bulges outward, or may be keel-shaped, or may present symmetrical protuberances. There is often marked increase in the transverse diameter of the head, and the lateral halves of the cranium often show marked asymmetry. The characteristic deformity of the face is that of the nose. In the first type the upper part of the nose seems to have fallen in, and the tip turns up; in the second, the lower portion of the nose is shortened, and appears in some way to be absorbed into the upper part. There are also less typical nasal deformities, as when the nose is flattened at the base or badly formed, the result of simple dystrophy, and not of necrosis. Protuberances and hyperostoses are found at the level of the epiphyses, especially noticeable at the upper extremity of the tibia and the lower extremity of the radius and ulna. But the tibia is the direct revelation of syphilis. Its form alone will reveal this disease. It is large, thick and misshapen, much widened and depressed in its middle portion. The crest is so much increased in size as to present a plane face. In general effect the bone is like the blade of a sword. This confirmation is never found in any condition other than hereditary syphilis, of which it is the pathognomonic sign. Chronic lesions of the testicle are of frequent occurrence. This, together with a history of middle ear trouble, interstitial keratitis, notched teeth, small scars upon the skin and mucous membrane, the imperfections of the bony skeleton, the arrest or anomalies of bodily development, and the special condition of the tibia, render the retrospective diagnosis of hereditary syphilis comparatively easy.—*N. Y. Medical Record.*

---

### LEWIS (B.) ON THE RÔLE OF THE POSTERIOR URETHRA IN CHRONIC URETHRITIS; CONCLUSIONS.

1. The causes usually given for the prolongation of cases of clap (presence or absence of gonococci, stricture of large calibre, the use of particular drugs in treatment, etc.) do not satisfactorily explain them, nor do they furnish reliable means for prognosticating the outcome of a case.

2. A singly widely prevalent cause for such prolongation of gonorrhea has, as yet, not proved its right to recognition as such.

3. Posterior urethritis, by reason of its anatomical seclusion and inaccessibility to ordinarily prescribed treatment, if frequent, offers the best explanation for such prolongation or repeated recurrence.

4. Scrutinizing clinical investigation shows posterior urethritis to be present in the majority of cases of prolonged or severe gonorrhea.

5. Direct, topical treatment to the posterior urethra is, therefore, necessary in the great majority of cases.

6. The causes usually given for producing posterior urethritis are not commonly found to be real factors in the clinic.

7. The mode of onset usually described does not coincide with that discerned in clinical observations.

8. These two latter observations confirm the probability that the posterior urethral infection is accomplished through the lymphatics, and explain the frequency of such infection.

9. Posterior urethritis is not a complication, but a natural phenomenon, of gonorrhea.—*N. Y. Med. Record,* July 29, 1893.

---

### Treatment of Sprained Ankle.

Dr. V. P. Gibney advocates (*The New York Polyclinic*) the treatment of sprained ankles by the use of strips of adhesive plaster. Dr. Gibney owes his indebtedness for the new method to a little book by Mr. Edward Cotterell, of the University College Hospital, London. It was not until the end of 1888 that the treatment advocated in brochure was fully digested and put into use by Dr. Gibney. He had all through his previous surgical career looked upon a sprain as a kind of mystery "not always so bad as a fracture, but sometimes more tedious," requiring fomentations for a little while, then a fixed dressing of plaster of Paris or silicate of sodium, crutches perhaps, and rest and massage afterward. He had never been attracted toward these methods, and he had come to expect a "stiffish" joint in nearly every case that came under his charge. His first case to be tried according to Cotterell's plan was that of a lady who had wrenched her right ankle severely. The usual external features of a sprain were present; no dislocation or fracture could be made out. Dr. Gibney first cut strips of rubber adhesive plaster about half an inch in width and long enough to completely encircle the foot. Then, with the foot well raised, he strapped it (the ankle) and the lower third of the leg with these strips, very much as if he had had an ulcer to treat. The first strip was carried over the outer side of the foot from near the base of the little toe. The second strip crossed the first, the third lapped over the first, and the fourth overlapped the second, and so on, until at the conclusion he had practically constructed a

Scultetus bandage of adhesive strips extending far enough to include the lower third of the limb. Over this he placed a cheese-cloth bandage to help the plaster strips to adhere to one another and to make the dressing more tidy. The patient was told to put on her stocking and shoe and to walk about the room. The walking was accomplished with some diffidence, but with no real difficulty. She was made to walk the next day and went out shopping without any bad results. The recovery was without relapse, and the usefulness of the ankle-joint was unimpaired.—*Kansas Med. Journal.*

---

### THE TREATMENT OF SUPPURATIVE CYSTITIS BY IODOFORM EMULSION.

Filippow (*Chirurgitsscheski Westnik,* 1892) reports very favorably the use of iodoform emulsion in chronic cystitis with suppuration. The bladder is first to be washed out with one-fourth per cent. solution of lactic acid, followed by from twenty to forty grammes of a ten per cent. iodoform emulsion, and allowed to remain for about fifteen minutes, and then drawn off. As high as nineteen injections may be made without danger. In none of the cases treated were any poisonous effects noticed. It is most important to see that the bladder is washed out before injecting the iodoform mixture.—*Univ. Med. Magazine, June,* 1893.

---

### SUBNITRATE OF BISMUTH IN EXTENSIVE BURNS.

Dr. Spigearny (*La Semaine Medicale,* No. 47, 1893) observed a case of extensive burn, involving four-fifths of the body and of the first degree. The case in question was a man who, while intoxicated in a Russian bathhouse, had a jet of superheated steam turned into the room where he was. Only his feet, legs, and the lower portions of his thighs were unaffected; all the rest of the body and the face presented one single scald covered with numerous bullæ and vesicles. Spigearny was called the sixth day after another physician had applied an iodoform dressing, which was completely soaked with the pus and serum from the lesions. His temperature was 40° C., and the urine contained no albumin. After having carefully washed the surface of the body by means of a boric acid solution and absorbent cotton, he dusted all the scalded surface over with the subnitrate of bismuth and applied over this a dressing of absorbent cotton filled with the same antiseptic and fixed the whole with tarlatan bandage. His face was simply dusted with the subnitrate. He prescribed besides this quinine and stimulants. The patient soon improved, his temperature fell, and at the end of a few days became normal. Complete recovery took place in three weeks, during which the dressing was renewed three times. He had no pain, neither did he present any signs of bismuth poisoning.—*Lancet-Clinic.*

---

### JOHNSON (R. W.) ON HEALING UNDER A MOIST BLOOD-CLOT IN ACCIDENTAL WOUNDS.

The writer calls renewed attention to the favorable way in which healing may take place if the wound cavity is allowed to fill with blood, and is then kept quiet without the use of drainage tubes.

Since many of his cases were traumatic, and not surgically clean when first seen, an additional interest is added.

The dressing was ordinarily left on about ten days,

He reports twelve cases:

Three gunshot wounds.

Two compound fractures of skull.

Two compound fractures of patella.

One punctured wound of knee joint.

One compound fracture of tibia.

One strangulated testicle.

One compound dislocation of elbow.

One amputation of both breasts.

The wounds healed kindly.

Many records of the successful use of the blood-clot have already come from Baltimore, and we are glad to know of the cases which Dr. Johnson reports.—*Ann. Surgery,* March 1, 1893.

